# Obstructive Sleep Apnea Is Associated with Worsened Hospital Outcomes in Children Hospitalized with Asthma

**DOI:** 10.3390/children11081029

**Published:** 2024-08-22

**Authors:** Jasmine Khatana, Aravind Thavamani, Krishna Kishore Umapathi, Senthilkumar Sankararaman, Aparna Roy

**Affiliations:** 1 Department of Pediatrics, Metro Health Medical Center, Case Western Reserve University School of Medicine, 2500 Metrohealth Dr, Cleveland, OH 44109, USA; jasmine.khatana@uhhospitals.org (J.K.); aroy@metrohealth.org (A.R.); 2 Division of Pediatric Gastroenterology, Hepatology and Nutrition, UH Rainbow Babies and Children’s Hospital, Case Western Reserve University School of Medicine, Adelbert Rd 2101, Cleveland, OH 44106, USA; senthilkumar.sankararaman@uhhospitals.org; 3 Division of Pediatric Cardiology, West Virginia University, Charleston Area Medical Center, 830 Pennsylvania Avenue, Charleston, SC 25302, USA

**Keywords:** obstructive sleep apnea, asthma, population based analysis, pediatric hopsitalization, mortality, mechanical ventilation

## Abstract

Background: Studies have shown a bidirectional relationship between asthma and obstructive sleep apnea (OSA). However, there is a paucity of national-level data evaluating the impact of OSA on hospital outcomes in pediatric hospitalizations for asthma. Methods: We analyzed the National Inpatient Sample and Kids Inpatient Database to include all pediatric hospitalizations with a primary diagnosis of asthma between 2003–2016. Using ICD codes, the pediatric asthma cohort was divided into two groups: those with and those without a concomitant diagnosis of OSA. The primary outcomes were in-hospital mortality and the need for mechanical ventilation. The secondary outcomes were the lengths of each hospital stay and total hospitalization charges. Results: We analyzed 1,606,248 hospitalizations during the 14-year study period. The overall prevalence rate of OSA was 0.7%. Patients with asthma and OSA were significantly older (8.2 versus 5.9 years) and were more often male, *p* < 0.001. The OSA group had several increased comorbidities. The overall mortality rate was 0.03%, and multivariate regression analysis showed that OSA was associated with 4.3 times higher odds of in-hospital mortality (95% CI: 2.4 to 7.6, *p* < 0.001). Furthermore, OSA was associated with a 5.2 times greater need for mechanical ventilation (95% CI: 4.8 to 5.5, *p* < 0.001). Linear regression analyses demonstrated that OSA independently contributed an additional 0.82 days to the hospital stay length (95% CI: 0.79 to 0.86, *p* < 0.001) and an extra 10,479 USD (95% CI: 10,110 to 10,848, *p* < 0.001) in hospitalization charges. Conclusion: OSA in children admitted with asthma is associated with poor hospital outcomes such as increased mortality risk, the need for mechanical ventilation, and increased healthcare utilization.

## 1. Introduction

Obstructive sleep apnea (OSA) is the most common form of sleep-disordered breathing, which means a total or partial collapse of the airways during sleep [1]. Adenotonsillar enlargement and obesity are the most important risk factors for pediatric OSA [2]. Asthma is the most common chronic respiratory disorder in the pediatric population. OSA and asthma often coexist and have many common comorbid conditions such as obesity, gastroesophageal reflux disease (GERD), and allergic rhinitis, among others [3,4].

Studies have shown a two-to-three-fold increased prevalence of OSA among patients with asthma [5,6,7]. Various studies have shown that there could be a bidirectional relationship between OSA and asthma, as OSA has been linked to poor asthma control, and, conversely, asthma predisposes patients to the development of OSA, making them an adverse combination, perpetuating disorders affecting overall health outcomes and quality of life [8,9,10]. The presence of OSA among patients with asthma is associated with uncontrolled severe respiratory disease, frequent exacerbations, and increased healthcare utilization [10,11,12,13,14]. In a recent meta-analysis, OSA was noted to be associated with severe asthma with decreased respiratory function, and conversely, asthma increased daytime sleepiness in OSA patients [15]. Furthermore, treatment of OSA has been shown to improve asthma control and quality of life among patients with asthma [16,17].

The interplay between OSA and asthma has mainly been attributed to local airway and systemic inflammation, along with snoring-induced possible damage of soft tissues leading to inflammation and edematous changes in the airway [18]. Patients with OSA are also noted to have elevated levels of inflammatory markers in exhaled breaths that are proportional to the severity of the disorder and also associated with altered innervation of the upper airway, which adversely affects the caliber of the airway, causing increased resistance and bronchospasm [19,20,21]. Thus, both OSA and asthma have been postulated to have a bidirectional adverse relationship. Despite the mounting evidence of this adverse relationship between OSA and asthma, there is a paucity of studies exploring this association in the hospitalized pediatric population. Thus, we hypothesize that the presence of OSA adversely impacts hospital outcomes among children and adolescents hospitalized with asthma. In this study, we aimed to evaluate the impact of OSA among children and adolescents admitted to the hospital with asthma.

## 2. Methods

We analyzed Healthcare Cost and Utilization Project (HCUP) databases—namely, the National Inpatient Sample (NIS) and the Kids Inpatient Database (KID)—to include all pediatric hospitalizations with a primary diagnosis of asthma. Both NIS and KID are administrative databases. NIS is the largest all-payer database containing data on inpatient hospitalizations in both the pediatric and adult populations, which are sampled at a rate of 20% from data covering more than 97% of the U.S population. The NIS database is released annually and contains the data from about 7 million hospital stays each year and is designed to provide national estimates. KID only contains data on the pediatric population, which are sampled at a higher rate (80%) compared to NIS and is released approximately once every 3 years. For this study, we utilized the KID database whenever available (2003, 2006, 2009, 2012, and 2016), and for the rest of the years, we used the NIS database.

All pediatric hospitalizations up to 18 years of age with a primary diagnosis of asthma were included in the study. Using the International Classification of Diseases (ICD) codes 9 and 10, the cohort was divided into two cohorts with and without a concomitant diagnosis of OSA: the OSA group and the control group, respectively. This methodology of using ICD in evaluating the relationship between OSA and asthma has been previously reported by other investigators [19]. We evaluated the data for various comorbid conditions such as obesity, GERD, allergic rhinitis, type two diabetes mellitus, dyslipidemia, and metabolic syndrome. The primary outcome variables were in-hospital mortality rates and the need for mechanical ventilation. Secondary outcome variables included the length of each hospital stay and total hospitalization charges in order to compare healthcare resource utilizations between the two groups. All data are deidentified as publicly available and are considered exempt from IRB approval.

## 3. Statistical Methods

The prevalence of OSA in patients with asthma during the study period was calculated utilizing both overall and annual data. The Shapiro–Wilk test was used to analyze normality, and a value of >0.05 was considered to indicate normal distribution. Categorical variables were compared using a Chi-square test and were expressed as frequencies and percentages. Continuous variables were compared using the student *t* test or Mann–Whitney U test (for parametric and non-parametric distribution, respectively) and expressed as the mean and standard deviation. A logistic regression analysis was used to explore the association between asthma and primary outcome variables (mortality and the need for mechanical ventilation). The results were expressed as odds ratio with 95% confidence interval. Separate linear regression models were constructed with the length of stay and total hospitalization charges as the outcome variables. The regression models were adjusted for potential confounding demographic factors and comorbidities. A *p*-value of <0.05 was considered statistically significant in our study. All statistical analyses were performed on SPSS software, version 25 (SPSS Inc., Chicago, IL, USA).

## 4. Results

We analyzed a total of 1,606,248 pediatric hospitalizations during the 14-year study period. The overall prevalence rate of obstructive sleep apnea was 0.7% (11,454). The prevalence rate increased from 0.4% in 2003 to approximately 1.6% in 2016, demonstrating a 4-fold increase during the study period, *p* < 0.001. Asthmatic patients with OSA were significantly older (8.2 versus 5.9 years) and more often male, *p* < 0.001 (Table 1). Compared to the control group, the OSA group had an increased proportion of African-American patients, and more than two-thirds of patients with OSA had public insurance, *p* < 0.001 (Table 1). The OSA group also had an increased prevalence of all the analyzed comorbidities, including obesity, type two diabetes, metabolic syndrome/dyslipidemia, allergic rhinitis, and GERD.

The overall mortality rate was 0.03% among all pediatric asthma-related hospitalizations. However, patients with OSA had an increased mortality rate compared to the control population (0.12% vs. 0.02%, *p* < 0.001). Similarly, the need for mechanical ventilation was significantly higher in the OSA group compared to the control population (10.2% vs. 1.4%, *p* < 0.001). Patients with OSA had longer durations of their hospital stays (3.3 ± 2.6 vs. 2.1 ± 1.6 days) and higher hospitalization charges (24,829 vs. 11,593) compared to patients without OSA, *p* < 0.001.

A multivariate logistic regression analysis showed that OSA was associated with 4.3 times higher odds of in-hospital mortality (95% CI: 2.4 to 7.6, *p* < 0.001) (Table 2). OSA was also adversely associated with asthma, but the other disorders such as allergic rhinitis, GERD, and type two diabetes mellitus were not associated. OSA was associated with a 5.2 times increased need for mechanical ventilation (95% CI: 4.8 to 5.5, *p* < 0.001) compared to hospitalized asthma patients without OSA (Table 3). Allergic rhinitis, GERD, obesity, and type two diabetes mellitus were also significantly associated with an increased risk of mechanical ventilation, whereas metabolic syndrome and dyslipidemia did not demonstrate an association with mechanical ventilation.

Linear regression analyses demonstrated that OSA independently contributed to an additional 0.82 days of hospital stay length (95% CI: 0.79 to 0.86, *p* < 0.001) and an extra 10,479 USD (95% CI: 10,110 to 10,848, *p* < 0.001) in hospitalization charges (Table 4).

## 5. Discussions

Our study highlights the increased prevalence of OSA in pediatric patients hospitalized with asthma, and the prevalence continued to increase during the study period. A concomitant presence of OSA in asthmatic patients was associated with adverse hospital outcomes and significantly higher overall healthcare expenditures.

The overall prevalence of OSA varies between 8 and 63% in the adult population with asthma [22,23,24]. In a recent meta-analysis, the estimated prevalence was 49.5% (95% CI: 36.39 to 62.6) [7]. In the pediatric age group, the prevalence rate of OSA has been reported to be as high as 66%; furthermore, the prevalence rate increased with the severity of the disease, being more prevalent in poorly controlled moderate to severe persistent asthma patients compared to mild or intermittent asthmatic patients [25]. Our study has an overall prevalence rate of 0.7%. In a similar nationwide population analysis using the NIS in the United States, the prevalence of OSA among hospitalized patients with asthma was estimated to be around 4.5% in the adult population [26]. These lower prevalence rates of OSA in hospitalized asthma patients using large national-level databases could be attributed to a lower proportion of coding by the clinicians leading to underestimation of true prevalence. Due to methodological limitations, we did not have access to the polysomnography results that might have provided us with the exact prevalence of OSA. Furthermore, our study brings attention to the steady increase in OSA over a 12-year period. This could be due not only to improving coding practices, but also to the simultaneously increasing general prevalence of risk factors of OSA, such as obesity [27,28,29,30].

Various studies have shown a bidirectional relationship between asthma and OSA [18,31,32]. Untreated OSA can impair the control of asthma, reduce the effectiveness of asthma medications, and increase the risk of exacerbations, as studies have shown the apnea–hypopnea index to be independently associated with asthma exacerbations [23]. Conversely, uncontrolled asthma may worsen OSA symptoms and reduce the efficacy of continuous positive airway pressure (CPAP) therapy [33]. Our study showed that children hospitalized with asthma and OSA had more than five times higher odds of requiring mechanical ventilation compared to those without OSA. Also, the presence of OSA was associated with a 4-fold increase in the risk of mortality among children admitted with asthma.

Becerra et al. demonstrated that the presence of OSA increased the length of stay of asthma-related hospitalizations by 7% and 14% in males and females, respectively [19]. Similarly, OSA contributed to 15.4% and 19.13% increases in hospitalization costs for males and females admitted with asthma, respectively [26]. Our study results concurred with this prior study, demonstrating that OSA contributed to 0.82 additional days of hospitalization and almost 10,500 USD extra in hospitalization charges. Thus, the early detection of OSA and appropriate management will help to improve clinical outcomes as well as decrease healthcare resource utilization.

Limitations: Our study has limitations to be acknowledged, as it was a database analysis involving administrative data. The inadvertent errors in the coding and billing should be considered while interpreting the results. As clinicians are more likely to code patients with severe OSA and omit patients with mild to moderate OSA, our data may have a certain bias with the omission of patients with mild to moderate OSA in the OSA group. We did not have access to data on clinical parameters such as the severity of the disease, apnea–hypopnea index, medication use, treatment details for OSA, and lab parameters. Further longitudinal data to evaluate for any causality, asthma control, and outpatient medication use, as well as the duration of OSA were not available for interpretation. We did have the reasons for the OSA diagnoses, such as enlarged adenoids and adenoids, neuromuscular disorders, genetic disorders, craniofacial diseases, children with hypotonia, obesity, etc. Each encounter in the database represents a unique hospitalization and not an individual patient; thus, patients with severe diseases may have been admitted multiple times and may have contributed to an overestimation of prevalence. Although we have utilized insurance as a surrogate for economic status, we do not have data on other socioeconomic factors such as median household income and education level that may have impacted disease control and outcomes.

Despite these limitations, our study has numerous strengths, including a significantly large sample size of nationally representative data over a 14-year time period in the pediatric age group. This large sample size enabled us to adjust for various confounders in our regression models that may be difficult to accomplish in single-center studies or studies with smaller sample sizes. Furthermore, the 14-year time period enabled us to objectively evaluate the increasing trend of OSA. All diagnoses utilized had specific codes in both ICD 9 and 10 classifications, thus making coding errors less likely and helping us to interpret the results with confidence.

## 6. Conclusions

Our study highlights the rising prevalence of OSA among hospitalized children with asthma. OSA in children with asthma adversely affected hospital outcomes by increasing the risk of mortality, the need for mechanical ventilation, and healthcare resource utilization. Our study findings emphasize the need to increase identification of these populations at risk of an adverse prognosis, as well as the need for more research into prompt treatment of OSA in order to improve hospital outcomes in children with asthma.

## Figures and Tables

**Table 1:** Comparison of demographics, clinical characteristics, and outcome measures between asthmatic patients with and without obstructive sleep apnea (OSA).

Parameters	Asthma Patients with OSA N = 11,454	Asthma Patients without OSA N = 1,594,794	*p* Value
Age (Mean ± SD)	8.2 ± 4.8	5.9 ± 4.3	<0.001
Gender			<0.001
Female	3937 (34.4%)	607,394 (38.4%)	
Male	7516 (65.6%)	973,612 (61.6%)	
Race			<0.001
Caucasian	2690 (23.5%)	469,735 (29.5%)	
African American	4405 (38.5%)	458,310 (28.7%)	
Hispanic	2316 (20.2%)	284,183 (17.8%)	
Others	2042 (17.8%)	382,346 (24%)	
Insurance			<0.001
Public	7924 (69.2%)	873,795 (54.8%)	
Private	2950 (25.8%)	602,920 (37.8%)	
Others	579 (5.1%)	117,859 (7.4%)	
Obesity	3872 (33.8%)	33,828 (2.1%)	<0.001
Type two diabetes mellitus	331 (2.9%)	2023 (0.12%)	<0.001
Metabolic syndrome	225 (2%)	593 (0.04%)	<0.001
Dyslipidemia	91 (0.8%)	862 (0.05%)	<0.001
Allergic rhinitis	604 (5.3%)	31,118 (2%)	<0.001
Gastroesophageal reflux disease	1917 (16.7%)	42,537 (2.7%)	<0.001
Mortality rate	14 (1%)	429 (0.02%)	<0.001
Need for mechanical ventilation	1169 (10.2%)	21,556 (1.4%)	<0.001
Duration of hospital stay in days (Mean ± SD)	3.3 ± 2.6	2.1 ± 1.6	<0.001
Total hospitalization charges in USD (Mean ± SE)	24,829 ± 415	11,593 ± 15	<0.001

**Table 2:** Logistic regression analysis of factors associated with mortality among children hospitalized with obstructive sleep apnea (OSA).

Parameters	Odds Ratio (95% CI)	*p* Value
Age	1.17 (1.15 to 1.19)	0
Gender Female vs. Male	0.84 (0.69 to 1.02)	0.081
Insurance		
Public	Ref	
Private	0.88 (0.71 to 1.08)	0.21
Others	1.26 (0.91 to 1.73)	0.16
Race/Ethnicity		
Caucasian	Ref	
African American	1.49 (1.18 to 1.89)	0.001
Hispanic	0.54 (0.25 to 1.17)	0.001
Others	0.55 (0.28 to 1.07)	0.94
Allergic Rhinitis	0.54 (0.25 to 1.17)	0.11
Gastroesophageal reflux disease	0 (0 to 0)	0.78
Obesity	0.43 (0.23 to 0.81)	0.008
Type two diabetes mellitus	0.84 (0.16 to 4.46)	0.83
Obstructive sleep apnea	4.36 (2.48 to 7.66)	<0.001

**Table 3:** Logistic regression analysis of factors associated with need for mechanical ventilation among children hospitalized with obstructive sleep apnea.

Parameters	Odds Ratio (95% CI)	*p* Value
Age	1.09 (1.09 to 1.09)	<0.001
Gender Female vs. Male	1.02 (1 to 1.05)	0.119
Insurance		
Public	Ref	
Private	0.82 (0.8 to 0.85)	<0.001
Others	0.77 (0.73 to 0.82)	<0.001
Race/Ethnicity		
Caucasian	Ref	Ref
African American	1.59 (1.53 to 1.64)	<0.001
Hispanic	1.23 (1.17 to 1.28)	<0.001
Others	1.34 (1.29 to 1.39)	<0.001
Allergic rhinitis	1.18 (1.09 to 1.28)	0.000
Gastroesophageal reflux disease	1.85 (1.75 to 1.96)	0.000
Obesity	1.33 (1.25 to 1.41)	0.000
Metabolic syndrome	0.83 (0.6 to 1.14)	0.244
Dyslipidemia	0.86 (0.6 to 1.23)	0.417
Type two diabetes mellitus	1.26 (1.06 to 1.51)	0.011
Obstructive sleep apnea	5.21 (4.87 to 5.58)	0.000

**Table 4:** Multivariate analysis evaluating impact of obstructive sleep apnea on hospital length of stay and hospitalization charges in children with asthma.

Parameters	Odds Ratio (95% CI)	*p* Value	Odds Ratio (95% CI)	*p* Value
Age	0.03 (0.03 to 0.03)	<0.001	287.6 (8651.03 to 294.52)	<0.001
Gender Female vs. Male	0.12 (0.11 to 0.13)	<0.001	518.35 (280.67 to 580.82)	<0.001
Race/Ethnicity				
Caucasian	Ref		Ref	
African American	−0.03 (−0.03 to −0.02)	0.36	1525.91 (455.88 to 1607.22)	<0.001
Hispanic	0.11 (0.10 to 0.12)	<0.001	4040.43 (1444.59 to 4132.92)	<0.001
Others	−0.02 (−0.03 to −0.01)	<0.001	−854.68 (3947.95 to −770.39)	<0.001
Insurance				
Public	Ref		Ref	
Private	−0.14 (−0.145 to −0.132)	<0.001	−445.28 (−938.98 to −378.79)	<0.001
Others	−0.14 (−0.15 to −0.126)	<0.001	−1270.63 (−511.76 to −1152.07)	<0.001
Allergic rhinitis	0.05 (0.03 to 0.07)	<0.001	1813.44 (−1389.19 to 2033)	<0.001
Gastroesophageal reflux disease	0.98 (0.97 to 1)	<0.001	6021.49 (1593.87 to 6206.57)	<0.001
Obesity	0.27 (0.25 to 0.29)	<0.001	1327.82 (2076.69 to 1550.53)	<0.001
Metabolic syndrome	0.48 (0.35 to 0.6)	<0.001	2743.74 (1105.1 to 4095.41)	<0.001
Dyslipidemia	0.08 (−0.04 to 0.19)	0.20	523.41 (1392.07 to 1768.45)	0.41
Type two diabetes mellitus	0.27 (0.2 to 0.35)	<0.001	−293.21 (−721.62 to 506.77)	0.47
Obstructive sleep apnea	0.83 (0.79 to 0.86)	<0.001	10,479.49 (−1093.18 to 10,848.08)	<0.001

## Data Availability

All data used in our study were gathered from publicy available database.
